# Role of Protein-Water Interface in the Stacking Interactions of Granum Thylakoid Membranes—As Revealed by the Effects of Hofmeister Salts

**DOI:** 10.3389/fpls.2020.01257

**Published:** 2020-08-14

**Authors:** Ottó Zsiros, Renáta Ünnep, Gergely Nagy, László Almásy, Roland Patai, Noémi K. Székely, Joachim Kohlbrecher, Győző Garab, András Dér, László Kovács

**Affiliations:** ^1^Institute of Plant Biology, Biological Research Centre, Szeged, Hungary; ^2^Neutron Spectroscopy Department, Centre for Energy Research, Budapest, Hungary; ^3^Laboratory for Neutron Scattering and Imaging, Paul Scherrer Institute, Villigen PSI, Switzerland; ^4^Institute for Solid State Physics and Optics, Wigner Research Centre for Physics, Budapest, Hungary; ^5^Neutron Scattering Division, Oak Ridge National Laboratory, Oak Ridge, TN, United States; ^6^Institute of Biophysics, Biological Research Centre, Szeged, Hungary; ^7^Jülich Centre for Neutron Science at MLZ, Forschungszentrum Jülich GmbH, Garching, Germany; ^8^Department of Physics, Faculty of Science, University of Ostrava, Ostrava, Czechia

**Keywords:** circular dichroism, granum, Hofmeister effect, thylakoid membranes, protein-water interface, small-angle neutron scattering, ultrastructure

## Abstract

The thylakoid membranes of vascular plants are differentiated into stacked granum and unstacked stroma regions. The formation of grana is triggered by the macrodomain formation of photosystem II and light-harvesting complex II (PSII-LHCII) and thus their lateral segregation from the photosystem I—light-harvesting complex I (PSI-LHCI) super-complexes and the ATP-synthase; which is then stabilized by stacking interactions of the adjacent PSII-LHCII enriched regions of the thylakoid membranes. The self-assembly and dynamics of this highly organized membrane system and the nature of forces acting between the PSII-LHCII macrodomains are not well understood. By using circular dichroism (CD) spectroscopy, small-angle neutron scattering (SANS) and transmission electron microscopy (TEM), we investigated the effects of Hofmeister salts on the organization of pigment-protein complexes and on the ultrastructure of thylakoid membranes. We found that the kosmotropic agent (NH_4_)_2_SO_4_ and the Hofmeister-neutral NaCl, up to 2 M concentrations, hardly affected the macro-organization of the protein complexes and the membrane ultrastructure. In contrast, chaotropic salts, NaClO_4_, and NaSCN destroyed the mesoscopic structures, the multilamellar organization of the thylakoid membranes and the chiral macrodomains of the protein complexes but without noticeably affecting the short-range, pigment-pigment excitonic interactions. Comparison of the concentration- and time-dependences of SANS, TEM and CD parameters revealed the main steps of the disassembly of grana in the presence of chaotropes. It begins with a rapid diminishment of the long-range periodic order of the grana membranes, apparently due to an increased stacking disorder of the thylakoid membranes, as reflected by SANS experiments. SANS measurements also allowed discrimination between the cationic and anionic effects—in stacking and disorder, respectively. This step is followed by a somewhat slower disorganization of the TEM ultrastructure, due to the gradual loss of stacked membrane pairs. Occurring last is the stepwise decrease and disappearance of the long-range chiral order of the protein complexes, the rate of which was faster in LHCII-deficient membranes. These data are interpreted in terms of a theory, from our laboratory, according to which Hofmeister salts primarily affect the hydrophylic-hydrophobic interactions of proteins, and the stroma-exposed regions of the intrinsic membrane proteins, in particular—pointing to the role of protein-water interface in the stacking interactions of granum thylakoid membranes.

## Introduction

In oxygenic photosynthetic organisms the light reactions of photosynthesis occur in the thylakoid membranes, which are densely packed with pigment-protein complexes of the two photosystems (PSII and PSI) and other constituents of the photosynthetic machinery. These membranes are organized in multilamellar systems, which evidently warrant efficient light capturing. During the more than 3 billion years of evolution, cyanobacteria and the chloroplasts of algae of different classes have evolved an astounding variation regarding the composition and ultrastructure of their thylakoid membranes (Solymosi, 2012; Solymosi and Keresztes, 2012).

Vascular plants evolved about 420 million years ago (Harrison and Morris, 2018). In their thylakoid membranes the PSII and PSI core complexes are associated with light-harvesting antenna complexes, LHCII and LHCI, respectively; these membranes also contain the cytochrome b6f complex and the ATP-synthase. A striking feature of their chloroplast ultrastructure is the differentiation of the thylakoid membranes into granum and stroma regions, also called stacked, or appressed, and unstacked, or non-appressed regions, respectively. The cylindrical grana stacks, of typically 10–20 layers with a diameter of 300–600 nm, are interconnected by single thylakoids of several hundred nm in length. Although PSII and LHCII reside mainly in the stacked membranes and PSI and the ATP synthase are predominantly found in the stroma thylakoids (Andersson and Anderson, 1980), the thylakoid membranes retain their continuity and enclose a single interior aqueous phase, the thylakoid lumen, and separate it from the outer, stroma aqueous phase (Mustárdy and Garab, 2003). Although many elements of this highly organized complex ultrastructure evolved earlier and can be recognized in green algae, the organization of the thylakoids into granum-stroma membrane assembly is a relatively recent and immensely successful product of the evolution (Gunning and Schwartz, 1999; Mullineaux, 2005). Its success is marked by the fact that vascular plants generate over 90% of the terrestrial photosynthetic productivity.

The tight appression of the thylakoid membranes in the grana ensures that chloroplasts contain an extremely large area-to-volume ratio, i.e. an optimized packing density of the membranes (Mustárdy and Garab, 2003; Dekker and Boekema, 2005). Evidently, this can only be warranted by a mechanism sorting the proteins according to their stroma-side protruding sizes. Whereas the protrusion of PSII and LHCII towards the stroma are small (<2 nm) (Daum et al., 2010), PSI and the ATP synthase, respectively, extend about 4 and 16 nm above the membrane surface (Miller and Staehelin, 1976; Ben-Shem et al., 2003). The lateral sorting of the complexes has been shown to be facilitated by cation-induced aggregation of PSII-LHCII supercomplexes; this step is followed by stacking of the adjacent membranes enriched in PSII-LHCII, which then stabilizes the structure (Garab et al., 1991; Garab and Mustardy, 1999; see also Garab, 2016). Evidence for the presence of PSII-LHCII macrodomains with long-range chiral order of the chromophores in the grana has been provided by circular dichorism (CD) spectroscopy (see Garab and van Amerongen, 2009; Garab, 2014; Tóth et al., 2016; Lambrev and Akhtar, 2019); ordered, semi-crystalline arrays of the supercomplexes have also been detected by electron microcopy techniques (Kouřil et al., 2012). Model calculations—using a two-dimensional matrix and cation-screened Coulomb interactions and van der Waals forces, dipole-dipole interactions, and lipid-induced protein-protein attractions—assigned the lateral segregation of the two photosystems to an interplay between electrostatic and lipid-mediated interactions (Rojdestvenski et al., 2002). With the stacking of PSII-LHCII domains in the grana regions, the interthylakoidal gap, accommodating the outer loops of two opposed PSII-LHCII supercomplexes, can be as low as 3.2 nm (Daum et al., 2010) or, in partially dehydrated thylakoid membranes, 2.6 nm (Kirchhoff et al., 2008).

Electron microscopy and tomography techniques revealed a highly organized interwoven structure of the granum and stroma thylakoid membranes, with the basic features of the stacked layers of granum thylakoids which are interconnected by stroma thylakoids wound around the grana in a quasi-helical fashion (Paolillo, 1970; Mustárdy and Garab, 2003; Mustárdy et al., 2008; Daum et al., 2010; Adam et al., 2011; Austin and Staehelin, 2011; Kowalewska et al., 2016). The most recent and highly elaborated electron tomography images have also uncovered that the entire granum-stroma thylakoid network is consolidated by arrays of right- and left-handed helical membrane structures (Bussi et al., 2019).

The robust granal ultrastructure suggests a high stability of the thylakoid membrane system, which nevertheless remains remarkably flexible in response to dynamically changing environmental conditions. Light-induced dark-reversible reorganizations have been shown to occur in isolated plant thylakoid membranes (Murakami and Packer, 1970; Garab et al., 1988; Nagy et al., 2011; Iwai et al., 2013) and in intact leaves (Ünnep et al., 2014). In general, different structural changes have been identified and linked to different short- and long-term regulatory mechanisms (Andersson and Anderson, 1980; Horton, 1999; Johnson et al., 2011; Nevo et al., 2012; Puthiyaveetil et al., 2016; Wood et al., 2018). Thus, granal thylakoid membranes evidently possess a high propensity to undergo well discernible structural changes. The plasticity of grana appears to be based on the inherent property of lipid:LHCII macroassemblies, which are capable to self-assemble into large arrays and to undergo reversible reorganizations induced by light and/or by subtle changes in the physico-chemical environment (Barzda et al., 1996; Zer et al., 1999; Simidjiev et al., 2000; Janik et al., 2013; Hind et al., 2014; Lambrev and Akhtar, 2019).

With regard to the physical mechanisms underlying the self-assembly and structural dynamics of the granum-stroma thylakoid membrane system, we can rely on many experimental observations and a few theoretical works. Most researchers agree that, in combination with van der Waals forces, electrostatic interactions, and particularly cations play key role in the stacking of membranes (see e.g. Garab and Mustardy, 1999; Chow et al., 2005; Dekker and Boekema, 2005; Daum and Kühlbrand, 2011). Indeed, it has been shown by electron microscopic studies that upon depletion of cations the granal structure is dismantled (Izawa and Good, 1966). The classical electrostatic theory of Gouy-Chapman has also been successfully applied to describe the surface-charge density of thylakoid membranes and its role in determining the structure and function of plant thylakoid membranes (Barber, 1982; Kana and Govendjee, 2016). The formation of large, chirally ordered lamellar aggregates of LHCII also depends on the proper electrostatic conditions (Simidjiev et al., 1997); and vice versa, the light-induced reversible reorganizations of lipid-LHCII macroarrays/membrane crystals were accompanied by release of cations (Garab et al., 2002; Hind et al., 2014). (For cation release of similar origin see Garab et al., 1998). Cation release associated with the functioning of the photosynthetic electron and proton transport has earlier been thoroughly documented (Hind et al., 1974).

Concerning the structural basis of stacking interactions in the grana, it has been shown that PSII-LHCII supercomplexes stack to each other *via* physical connections of specific N-terminal regions of the light-harvesting complexes that span the stromal gap—possibly through salt bridges between negatively-charged amino acid residues (Albanese et al., 2020). This model elegantly explains the stacking of granum membranes in the presence of the most abundant, so-called C_2_S_2_M PSII-LHCII supercomplexes in plants. It must, however, be noted that stacking could also be induced, by increasing the cation concentration, after large sections of the outer loop segments of thylakoid proteins had been digested by trypsin (Jennings et al., 1981). Also, chiral macrodomains can be generated in thylakoids of the chlorophyll *b*-less chlorina mutants, deficient in LHCII, but it requires considerably higher concentrations of Mg^2+^ than in the wild type; further, the concentration of osmoticum also had to be increased in the absence of LHCII (Garab et al., 1991). Further, the thermal stability of the chiral macrodomains, characteristic of the macro-organization of the protein complexes in the grana, have been shown to depend both on the ionic and osmotic strengths of the medium (Cseh et al., 2000) and on the lipid composition of membranes (Krumova et al., 2010) as well on the growth-light intensity of plants (Petrova et al., 2019).

In broad terms, the structural dynamics of grana can be explained within the frameworks of a theory explaining grana stacking *via* an interplay between repulsive electrostatic and hydrostructural and attractive, van der Waals forces (Puthiyaveetil et al., 2017). It is shown that variations in the electrostatic forces, which might be modulated by ionic movements (see above) or by the phosphorylation of LHCII or other phosphoproteins, affect both the lateral organization and stability of stacking. Other theoretical calculations have also shown that charge movements exert very strong effect on stacking interaction of membranes, and thus on the structural dynamics of grana (Majee et al., 2019). Within the frameworks of a theoretical model, investigating the effect of Mg^2+^ on the entropy of the system, it has been proposed that the underlying physical mechanisms might be a combination of several events: i) the attraction between discrete, oppositely-charged areas of grana; ii) the release of loosely-bound water molecules from the interthylakoidal space; iii) variations in the orientational freedom of water dipoles; and iv) the lateral rearrangements of membrane components (Jia et al., 2014).

The major aim of our work presented here is to obtain new insight on the forces involved in the self-assembly and dynamics of the granum-stroma thylakoid membrane system. To this end, we tested the effects of Hofmeister salts on the organization of pigment-protein complexes and the ultrastructure of thylakoid membranes. Contrary to cations, mobile anions are usually not considered exerting a direct effect on the complex organization of thylakoid membranes; however, they are well-known to have a determining role in Hofmeister effects (Lo Nostro and Ninham, 2012). Hofmeister effects are related to the ability of neutral salts, of moderate and high-salt concentrations (>100 mM), to modify the aggregation and crystallization properties of proteins (and colloid particles, in general), as well as to affect protein structure, dynamics and function (Collins and Washabaugh, 1985). Salts that facilitate aggregation and stabilize closed protein conformations, as in the most common native cases, are called kosmotropes, e.g., (NH_4_)_2_SO_4_, NaF, Na-acetate; while the structure-destabilizing salts are called chaotropes, e.g., NaBr, NaClO_4_, NaSCN. At the boundary between the two groups, NaCl is considered to be “Hofmeister-neutral”, or slightly chaotropic. Note that Hofmeister effects are dominated by anions but, according to their similar effects, cations can also be arranged to a Hofmeister series, as well as non-ionic compounds such as polyols (Cacace et al., 1997).

Since kosmotropic ions are also shown to be “water structure makers”, while chaotropes are “water structure breakers” (Robinson and Jencks, 1965), it has been generally believed that Hofmeister effects are mediated by water structure at the interacting interfaces (Melander and Horváth, 1977; Collins and Washabaugh, 1985), although a coherent theory was missing until 2007, when a phenomenological formalism using the protein-water interfacial tension, as a key parameter, could qualitatively account for the diversity of manifestations of Hofmeister effect (Dér et al., 2007). Based on the formal match of the theory with that of hydrophobic effects (Tanford, 1979), Hofmeister effects could be interpreted as a modification of hydrophobic/hydrophilic interactions, described by the surface terms of the Gibbs free energy. Since then, a row of experimental and theoretical evidences supporting the predictions of the theory have been published (Khoroshyy et al., 2013; Szalontai et al., 2013; Bogár et al., 2014; Násztor et al., 2016; Násztor et al., 2017; Kovacs et al., 2018).

Here, we investigated the effects of chaotropic salts on the complex structure of chloroplast thylakoid membranes by structure-sensitive experimental techniques—transmission electron microscopy (TEM), CD spectroscopy and small-angle neutron scattering (SANS)—in order to reveal the possible importance of hydrophobic/hydrophilic interactions in maintaining the hierarchical organization of these paradigmatic structural units of the photosynthetic energy-transducing machinery of plants. The two non-invasive techniques, CD spectroscopy and SANS are sensitive to the macro-organization of the protein complexes and of the thylakoid membranes, respectively.

CD spectroscopy in the visible range provides information on hierarchically organized molecular assemblies (Garab and van Amerongen, 2009). Ordered arrays of the pigment-protein complexes, such as the PSII-LHCII macrodomains, give rise to giant, psi-type CD (CDψ) signal (psi, polymer or salt induced), due to long-range interactions of the chromophores, whereas the pigment-protein complexes display excitonic CD signals arising from short-range interactions between pigment dipoles. SANS carries information on the periodicity and repeat distances (RDs) of multilamellar thylakoid membranes, averaged for the entire volume of sample exposed to the neutron beam (Nagy and Garab, 2020).

We show that chaotropic salts gradually break down the structure of granum thylakoids, contrary to their kosmotropic and Hofmeister-neutral counterparts which do not have substantial effects. The time-evolution of dismantling follows the order of descending hierarchical complexity, while the kinetics and extent of structural decline correspond to the position of chaotropic ions in the Hofmeister series. These findings are interpreted as a consequence of tousling the ordered intergranal water layer by the chaotropic agents. The results call the attention to the fundamental role of water molecules between adjacent thylakoid membranes, both in maintaining stability of and providing flexibility for granum stacks, the highly organized ultrastructures in chloroplasts.

## Material and Methods

### Plant Materials and Isolation of Thylakoid Membranes

Pea, barley and tobacco plants were grown in greenhouse at 22°C under natural light conditions. Thylakoid membranes were freshly isolated from 3-week-old pea (*Pisum sativum* sp. Rajnai törpe), 2-week-old wild-type and chlorina mutant barley (*Hordeum vulgare*) and 2.5‑3-months-old tobacco (*Nicotiana tabacum* L. cv. Petit Havana SR) leaves, as described earlier (Zsiros et al., 2019). Leaves were homogenized in buffer A (50 mM Tricine-KOH pH 7.5, 0.4 M sorbitol, 5 mM MgCl_2_, 5 mM KCl). The homogenate was filtered through four layers of cheesecloth and the supernatant was centrifuged for 2 min at 300×*g*. After the centrifugation, the supernatant was further centrifuged at 5,000×*g* for 10 min. The pellet was resuspended in the hypotonic buffer B (50 mM Tricine-KOH pH 7.5, 5 mM MgCl_2_, 5 mM KCl). After a short, 5–10 s, osmotic shock, breaking the envelope membrane, the osmolarity was returned to isotonic conditions by adding equal volume of double-osmotic medium (50 mM Tricine-KOH pH 7.5, 0.8 M sorbitol, 5 mM MgCl_2_, 5 mM KCl) and the suspension was centrifuged at 5,000×*g* for 10 min. The pellet containing intact thylakoid membranes were resuspended in buffer A and used for further measurements. All steps of the isolation were performed at 4°C and in a dim light. Light-harvesting complexes were purified from pea leaves as previously described (Simidjiev et al., 1997).

### Circular Dichroism Spectroscopy

CD spectra were measured on a Jasco J-815 CD spectropolarimeter at room temperature with a bandwidth of 2 nm and data pitch of 1.0 nm. The scan speed was set to 100 nm/min and the integration time was 1 s. Simultaneously with the CD spectra, absorption spectra were recorded as well and the CD spectra were normalized to the red absorbance maxima, at around 680 nm. 750 nm reference wavelength was used to determine the amplitude of the (+)690, (-)675 and (+)505 CDψ bands. The chlorophyll concentration of the samples was 30 µg/ml. Three to five independent biological replicates were measured. The exact number is indicated in the *Figure Legends*.

### Specimen Preparation for Electron Microscopy

Thylakoid membranes were fixed in Karnovsky solution containing 2% paraformaldehyde (Sigma; St. Louis, MO, United States) and 2.5% glutaraldehyde (Polysciences; Warrington PA, United States) in phosphate buffer for overnight at 4°C. After fixation, the samples were rinsed in distilled water (pH 7.4) for 10 min followed by a 2% osmium tetroxide (in distilled water, pH 7.4) solution for 60 min. After osmification, the samples were briefly rinsed in distilled water for 10 min, then dehydrated through a graded series of ethanol (from 50% to 100%; Molar; Halasztelek, Hungary) for 10 min in each concentration and proceeded through propylene oxide. Dehydrated samples were embedded in an epoxy-based resin (Durcupan ACM; Sigma), then polymerized at 56°C for 48 h. 50 nm ultrathin sections were cut from the resin blocks on an Ultracut UCT ultramicrotome (Leica; Wetzlar, Germany) and samples were mounted on single-hole formvar-coated copper grids (Electron Microscopy Sciences; Hatfield, PA, United States). Ultrathin sections were contrasted with 2% uranyl acetate (Electron Microscopy Sciences) in 50% ethanol (Molar) and 2% lead citrate (Electron Microscopy Sciences) in distilled water. Samples were systematically screened at 5,000× magnification to localize the membranes on the grid. Afterwards, images of thylakoid membranes were recorded at 10,000‑20,000× magnification with a 16 MP Matataki Flash scientific complementary metal–oxide–semiconductor (sCMOS) camera (JEOL).

### Small-Angle Neutron Scattering (SANS)

The experiments were performed on the SANS I instrument of the Paul Scherrer Institute (PSI, Villigen, Switzerland). The applied settings for the measurement of the samples were: SD, 11 m; collimation, 15 m; λ, 6 Å. (SD, sample-to-detector distance; λ, neutron wavelength). Isolated thylakoid membranes of about 1 mg/ml chlorophyll content, suspended in ^2^H_2_O-based buffer A (Nagy et al., 2011), were filled in quartz cuvettes with 2 mm path length and were aligned in a magnetic field, using permanent magnets of ~ 0.4 T field strength, providing a magnetic field in the horizontal direction perpendicular to the neutron beam. Data obtained with pea, spinach and tobacco thylakoid membranes were qualitatively similar to each other but tobacco displayed the most intense Bragg peak, and thus tobacco thylakoids were used for quantitative analysis. All experimental data were corrected for detector efficiency and normalized to the number of incoming neutrons; the instrumental background, recorded with the beam blocked by cadmium was subtracted. For data treatment, the “Graphical Reduction and Analysis SANS Program” package (GRASP) (developed by C. Dewhurst, ILL) was used. The recorded 2D scattering signal was radially averaged in two sectors with 75° opening angle, and the scattering intensity (I) was plotted as a function of the scattering vector, Q, and used for further analyses. RD was calculated using the formula RD=2π/Q*, where Q* is the position of the Bragg peak (Nagy and Garab, 2020).

Time resolved measurements were carried out to survey the structural changes in isolated thylakoid membranes upon exposure to different concentrations of NaSCN, NaCl, and NaClO_4_, using 5 M stock solutions and different volumes of ^2^H_2_O-based buffer A, in order to obtain the same dilution. To minimize the dead-time (to about 1 min), the salt solutions were added directly into the cuvettes containing the untreated control.

Three independent biological replicates were measured.

## Results

**Figure 1:**
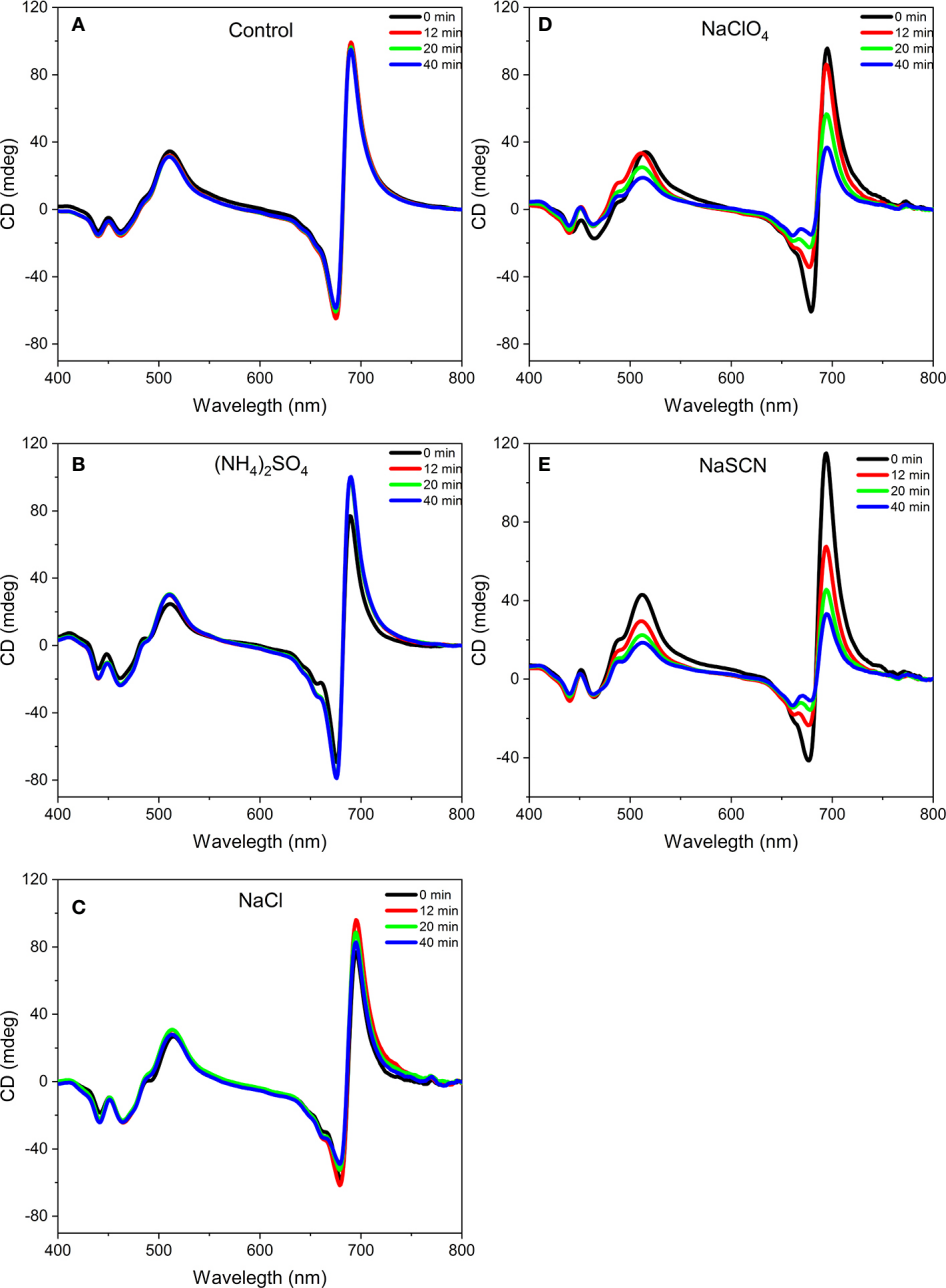
Representative circular dichroism spectra of isolated pea thylakoid membranes without treatment **(A)** and before (0 min) and after (12, 20, and 40 min) of their treatments with 1 M (NH_4_)_2_SO_2_ **(B)**, 1 M NaCl **(C)**, 1 M NaClO_4_ **(D)**, and 1 M NaSCN **(E)**. The spectra are normalized to the corresponding red absorbance maxima [OD(680 nm)=1.0] of the untreated samples. Note that the 12, 20, 40 min traces in **(A)**, and the 0 and 12 min traces in **(B)**, largely overlap. Five independent biological replicates were measured and a typical data set is shown.

The effect of Hofmeister salts on the macro-organization of pigment-protein complexes of thylakoid membranes was investigated by CD spectroscopy. Thylakoid membranes isolated from pea were treated with salts ranked at different positions of the Hofmeister series at various concentrations, and the changes in the amplitudes were followed as a function of time. In untreated membranes, the CD spectra are dominated by CDψ bands, originating from long-range interactions in chirally organized PSII-LHCII macrodomains, peaking at around (+)690, (-)675, and (+)505 nm (Garab and van Amerongen, 2009, see also **Figure 1**).

**Figure 2:**
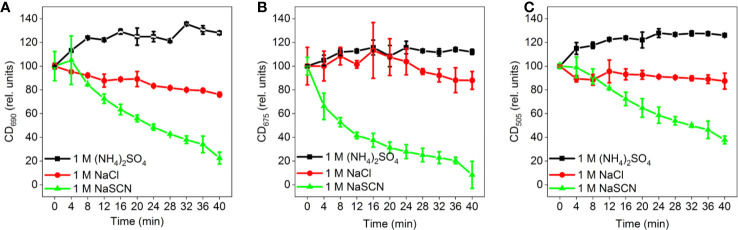
Time-courses of the effects of different Hofmeister salts on the amplitudes of the different CD_ψ_ bands of isolated pea thylakoid membranes: **(A)** (+)690 CD_ψ_, **(B)** (-)675 CD_ψ_, and **(C)** (+)505 CD_ψ_; the corresponding CD amplitudes were determined with reference to the 750 nm signals. The membranes were treated at t= 0 min with 1 M (NH_4_)_2_SO_4_, 1 M NaCl, and 1 M NaSCN, as indicated. Data points are representing the mean values of five independent biological replicates ± SD.

The kosmotropic salt, (NH_4_)_2_SO_4_, did not induce any observable changes in the CD spectrum at 1 M concentration (**Figure 1B**). At the same concentration, NaCl, regarded as a Hofmeister neutral salt, caused very slight decrease in the psi-type CD bands (**Figure 1C**) while weaker and stronger chaotropic agents, NaClO_4_ (**Figure 1D**) and NaSCN (**Figure 1E**), respectively, diminished the CDψ signals, with more pronounced effect observed with NaSCN. At the same time, no significant effects were observed on the excitonic bands at around (‑)650 nm and between 400 and 460 nm. (Some losses in the excitonic band-pair of CD_480-470 nm_ originate from the monomerization of LHCII trimers (Garab and van Amerongen, 2009). The time courses of the CD-changes at different wavelengths upon chaotropic treatment showed that the different CDψ amplitudes decreased at different rates (**Figure 2**). The fastest rate was observed for the (-)675 nm band while the (+)690 and (+)505 nm bands exhibited a significantly slower kinetics.

**Figure 3:**
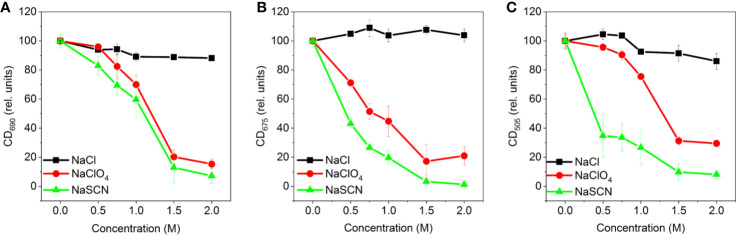
Concentration dependences of the effects of different Hofmeister salts (NaCl, NaClO_4_, and NaSCN) on the amplitudes of CD_ψ_ bands of isolated pea thylakoid membranes: **(A)** (+)690 CD_ψ_, **(B)** (-)675 CD_ψ_ and **(C)** (+)505 CD_ψ_; the amplitudes, relative to the control, were determined 20 min after the treatment, with reference to the corresponding 750 nm signal. Data points are representing the mean of three independent biological replicates ± SD.

The concentration dependence of chaotropic salts was also in good agreement with their rank in the Hofmeister series. As shown in **Figure 3**, NaCl exerted only marginal effects on the CD signals, whereas NaSCN proved to be stronger than NaClO_4_.

**Figure 4:**
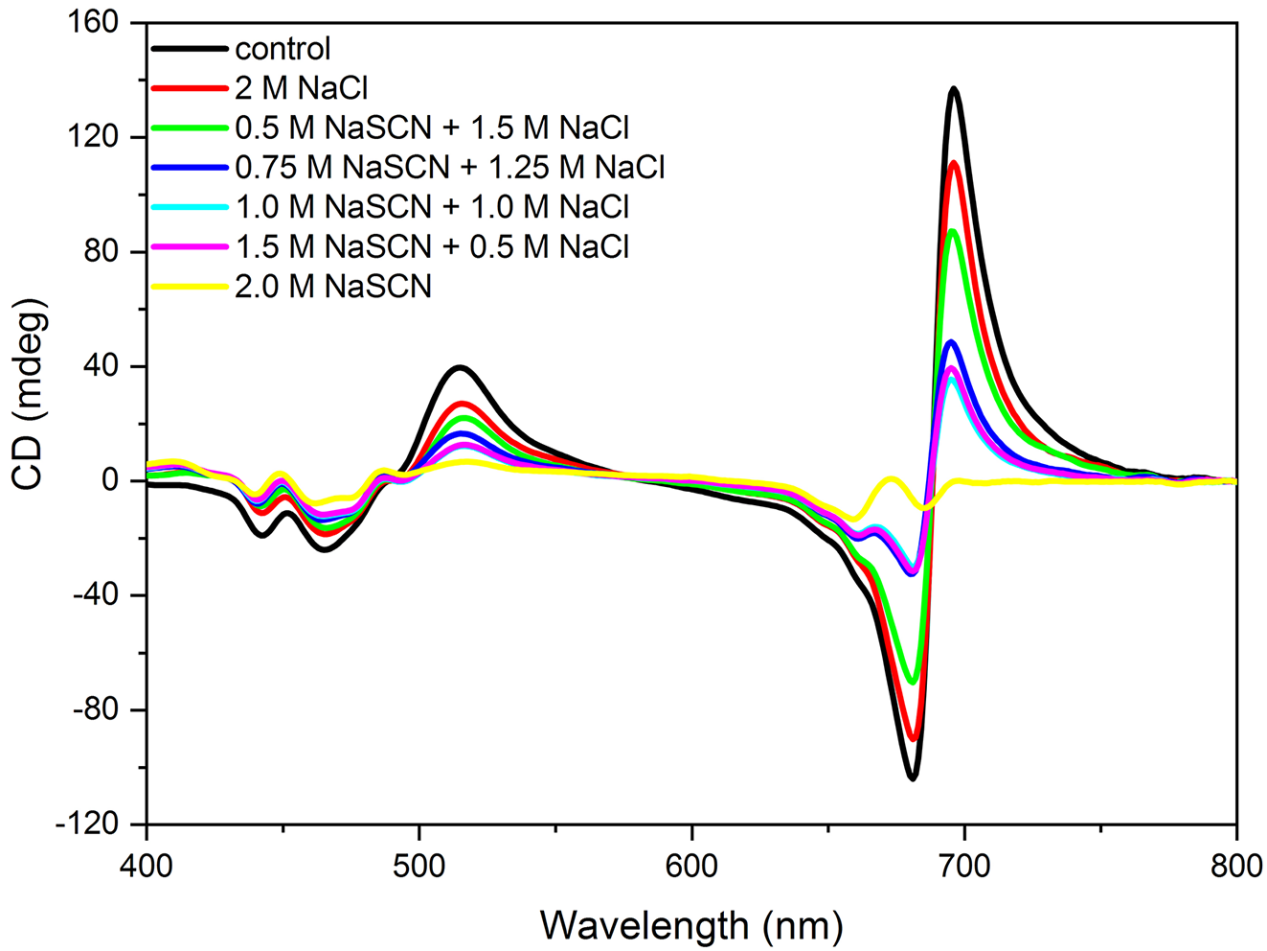
CD spectra of isolated pea thylakoid membranes in the absence (control) and presence of 2 M Hofmeister-salt combinations composed of NaCl and NaSCN—with constant Na^+^ concentration and different concentrations of Cl^-^ and (SCN)^-^, as indicated. The spectra were recorded 20 min after the treatments. The spectra are normalized to the corresponding red absorbance maxima [OD(680 nm)=1.0] of the untreated samples. Three independent biological replicates were measured and a typical data set is shown.

It has been shown that ionic strength and osmotic potential has a role in the macro-organization of thylakoid membranes, detected by CD spectroscopy (Garab et al., 1991) and SANS measurements (Posselt et al., 2012). To assess their possible contribution to the chaotropic-salt induced changes, we recorded CD spectra of isolated thylakoid membranes exposed to different mixtures of NaCl and NaSCN, in which the ionic strength, osmotic potential and sodium ion concentration were kept constant (**Figure 4**). The gradual exchange of Cl^-^ to SCN^-^ led essentially to the same changes in the CD spectrum as NaSCN alone, indicating that the alterations in the thylakoid macro-organization were caused mainly by the Hofmeister effect of the SCN^-^ anion.

**Figure 5:**
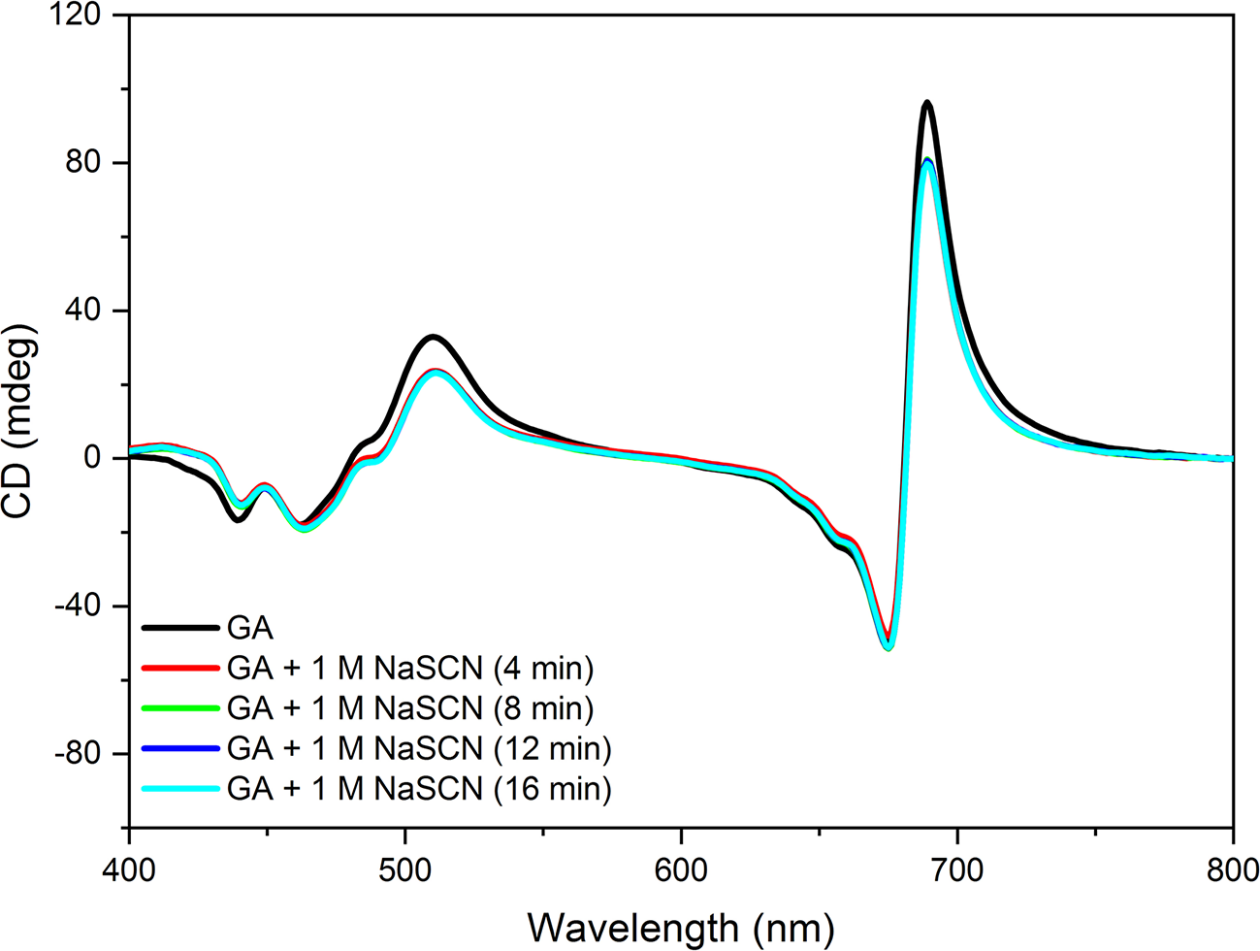
CD spectra of isolated pea thylakoid membranes fixed for 1 min with 2% glutaraldehyde (GA) and spectra of GA-fixed membranes treated with 1 M NaSCN and recorded at different times after the GA fixation. The spectra are normalized to the red absorbance maximum [OD(680 nm)=1.0] of the GA-treated sample. Note that the 4, 8, 12, and 16 min traces overlap. Three independent biological replicates were measured and a typical data set is shown.

**Figure 6:**
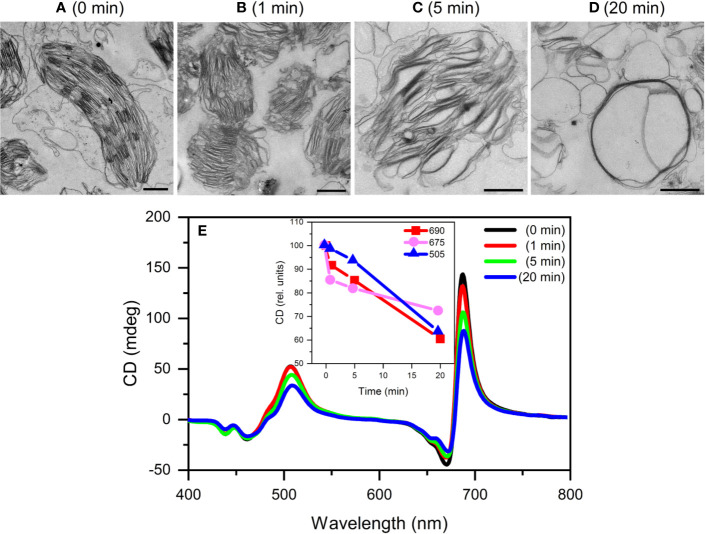
Typical TEM images **(A–;D)** and CD spectra **(E)** of untreated (0 min) and 0.5 M NaSCN treated isolated pea thylakoid membranes obtained after different time intervals following the treatment at t=0 min. The treatments were halted at 1, 5 and 20 min by 2% glutaraldehyde and the membranes were then used for TEM and CD experiments. Inset in **(E)**, time course of the variations in the different CD_ψ_ bands. Scale bars in **(A**–**D)**, 1 μm.

In order to test the chaotropic effect on membrane stacking, and to compare TEM data with CD, aliquots were taken at 0, 1, 5, and 20 min after the addition of 0.5 M NaSCN to the membranes; these aliquots were fixed immediately with 2% glutaraldehyde. By applying time-series CD measurements on glutaraldehyde-treated thylakoid membranes, we proved that glutaraldehyde fixation efficiently prevented the effect of NaSCN (**Figure 5**). Glutaraldehyde-fixed untreated and NaSCN-treated samples were subjected to further conventional fixation for subsequent TEM analysis (**Figure 6**). TEM images revealed well-defined intact grana in the control samples (**Figure 6A**), which were largely disorganized already 1 min after the addition of NaSCN (**Figure 6B**). After 5 min (**Figure 6C**), the presence of grana could not be discerned but membranes with tight stacking were still present. After 20 min (**Figure 6D**), virtually only single membranes remained, which formed large vesicles, some of which appeared to contain stacked membrane regions. As shown in **Figure 6E**, the CDψ bands persisted longer than the well-defined granal ultrastructure. After 20 min of NaSCN addition, more than 60% of the CDψ bands were still present, when the grana had already disappeared (cf. **Figure 6D**).

**Figure 7:**
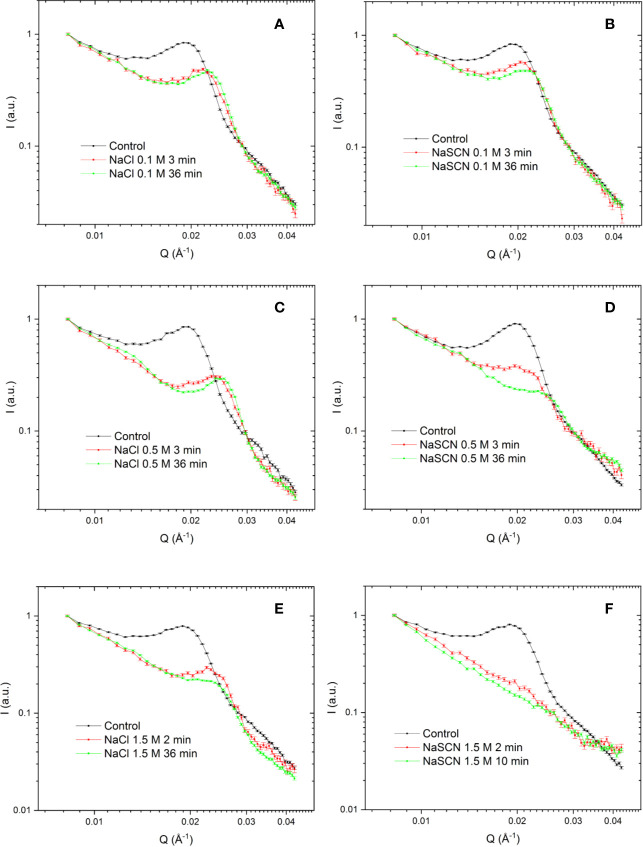
SANS profiles of isolated tobacco thylakoid membranes in the absence (control) and presence of different concentrations of NaCl **(A**, **C**, **E)** and NaSCN **(B**, **D**, **F)** recorded at different times after the addition of salts. To optimize the S/N of the SANS curves, different data acquisition and averaging times (1‑5 min) were applied, dictated by the rate of structural changes induced by NaSCN. The time-labels mark the midtimes between the start and the end of data acquisition. For better comparison all curves are normalized to 1 at Q = 0.00832 Å^-1^. Three independent biological replicates were measured and a typical data set is shown.

In order to determine the chaotropic effect on the periodic organization of the thylakoid membranes, SANS experiments were performed. The effects of NaCl and NaSCN on the membrane organization were compared with each other at 0.1, 0.5, and 1.5 M concentrations, and with the untreated sample (**Figure 7**). Control thylakoid samples exhibited scattering curves with a characteristic diffraction peak, Q*, at around 0.2 Å^-1^ (Nagy et al., 2011). Via fitting the curves with the sum of a power function and a Gaussian (see Nagy et al., 2013), the RD of the thylakoid membranes was estimated to be 332 ± 2 Å (mean value ± SD from eight samples). This value is somewhat larger than the RD values obtained earlier by SANS and EM under similar experimental conditions (Ünnep et al., 2014). Upon the addition of 0.1 M NaCl, the characteristic peak shifted to higher momentumtransfer values. Similar shrinkage has earlier been reported upon increasing the concentration of the osmoticum (Posselt et al., 2012) and upon replacing sorbitol for NaCl in the suspension medium (Ünnep et al., 2014). The structural changes induced by the addition of 0.1 M NaCl were essentially completed on the time-scale of few minutes (**Figure 7A**). At 0.1 M, the effect of NaSCN treatment was very similar to that of NaCl (**Figure 7B**). This suggests that, at this relatively low concentration, the structural changes are predominantly induced by a cationic effect—leading to a decrease in the RD of the thylakoid membranes to between about 280 and 290 Å. Indeed, high concentrations of monovalent cations, in the range of some hundred millimolar concentrations, have been shown to induce stacking (Izawa and Good, 1966; Hind et al., 2014). It was also interesting to note that both with NaCl and NaSCN the integrated Bragg-peak intensities decreased to very similar extents, to about 50% compared to the control. This may originate from some contrast-losses due to a narrowing of the interthylakoidal space in the grana.

At 0.5 M, NaCl induced some further shrinkage of the thylakoid membranes to about 260 Å, a value which was reached in a few minutes (**Figure 7C**). In contrast, at this concentration, the chaotropic effect of NaSCN became clearly discernable (**Figure 7D**): in addition to the shrinkage, the amplitude of the Bragg peak dramatically decreased compared to the NaCl-treated sample and the peak was gradually smeared—a clear sign of the gradual loss of the periodic order of the thylakoid membranes. Also, NaClO_4_ treatments caused a similar, gradual loss in the Bragg peak (data not shown), corroborating the notion that deteriorations of the membrane ultrastructure were caused by Hofmeister effect. With 1.5 M NaCl, while sufferred some further scattering intensity losses, the periodic order of the thylakoid membranes was largely retained (**Figure 7E**). In contrast, with 1.5 M NaSCN the decay of the periodic membrane ultrastructure was spectacularly accelerated; it was lost in less than 5 min (**Figure 7E**). It is to be noted here, that these agents, including even NaCl, might have also induced changes in the microscopic structure of the membranes, affecting their form factor and thus their scattering length distributions, which could, in principle, be analyzed within an advanced mathematical model recently elaborated for cyanobacteria (Jakubauskas et al., 2019). This would require a detailed and systematic approach, paying also attention to the substantial differences between the ultrastructure of thylakoid membranes in cyanobacteria and higher plants; this is outside the scope of the present study.

**Figure 8:**
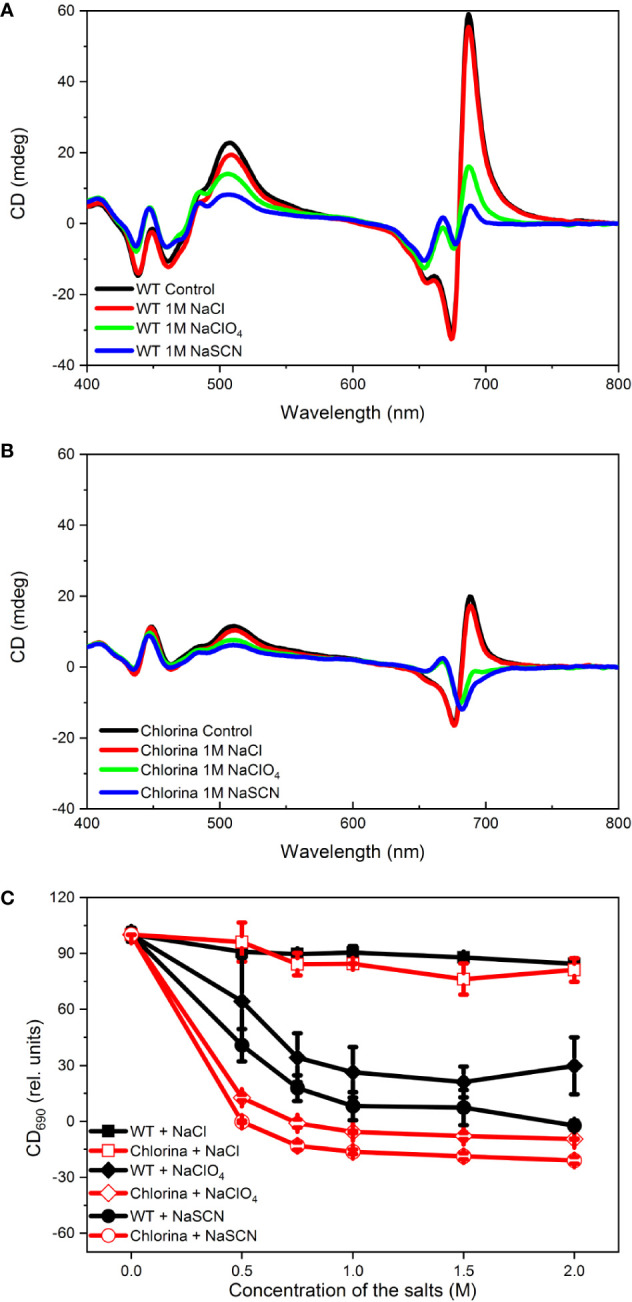
Concentration dependence of the effects of NaCl, NaClO_4_ and NaSCN on the CD spectra of WT **(A)** and Chl-b-less (Chlorina) mutant **(B)** barley thylakoid membranes. The amplitudes of (+)690 CD_ψ_ **(C)**, relative to the untreated control were determined 60 min after the addition of salts. Data points are representing the mean of 5 independent biological replicates ±SD.

In order to test if and how much the Hofmeister effects depend on the macro-organization of the protein complexes, and on the LHCII content of grana, in particular, we performed experiments on wild type and chlorophyll-b-less, chlorina-f2 mutant of barley. This mutant has been reported to lack LHCs but to retain the granal structures of WT plants (Goodchild et al., 1966). The CD spectra of detached leaves of the chlorina-f2 mutant has been shown to possess relatively strong (+)690 and (−)674 nm CDψ bands, but weak (+)506 nm band, indicating a different type of lateral organization of PSII supercomplexes (Tóth et al., 2016). A comparison of CD spectra of thylakoid membranes isolated from the wild type and mutant leaves in an earlier study revealed that the generation of the psi-type bands required considerably higher ionic strength to screen the negative repulsive forces, and higher concentration of the osmoticum than in the wild type (Garab et al., 1991). In accordannce with these data, compared to the wild type thylakoids (**Figure 8A**), chlorina-f2 membranes displayed weaker psi-type CD bands (**Figure 8B**). We also observed that the (+)690 nm CDψ of thylakoid membranes isolated from chlorina-f2 mutant exhibited higher sensitivity to chaotropic agents than the wild-type membranes (**Figure 8C**); data in **Figure 8** also demonstrate that Hofmeister salts on barley thylakoid membranes exert similar effects as in pea. Further, these data support the notion (Tóth et al., 2016) that LHCII plays a major role in the stabilization of the macro-organization of protein complexes in the thylakoid membranes.

**Figure 9:**
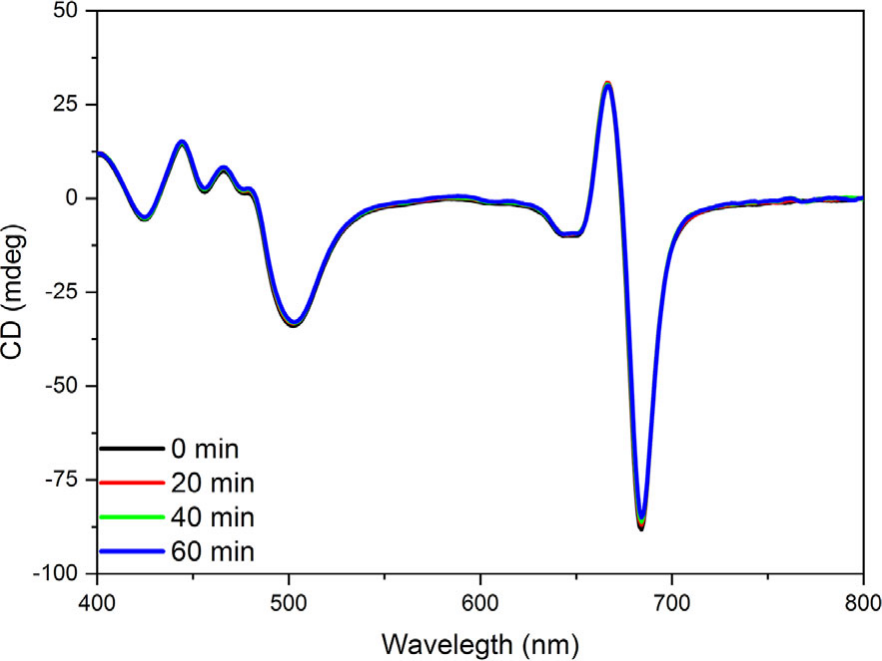
CD spectra of untreated (0 min) and 2 M NaSCN treated LHCII microcrystals at different times after the start of the treatment, as indicated. The spectra are normalized to the red absorbance maximum [OD(680 nm)=1.0] of the untreated samples. Note that the 0, 20, 40, and 60 min traces overlap. Three independent biological replicates were measured and a typical data set is shown.

We also investigated the Hofmeister effect on the structure of LHCII itself. Isolated LHCII microcrystals were exposed to 2 M NaSCN. The presence of CDψ at (-)684 nm in these tigthly stacked lamellar aggregates of isolated LHCII indicates the long-range order of the chromophores, warranted by the ordered array of LHCII apoproteins (Miloslavina et al., 2012). Neither the excitonic bands, originating from the short range pigment-pigment interactions within LHCII subunits, nor the CDψ exhibited significant changes during the 1-h long incubation time, showing that NaSCN has no chaotropic effect on LHCII itself (**Figure 9**).

## Discussion

Understanding the physical mechanisms of the interactions stabilizing the complex, hierarchical structure of plant chloroplast thylakoid membranes is of pivotal importance in photosynthesis research. The role of electrostatic interactions (both cation-mediated attractive, and the repulsive forces) as well as the van der Waals forces (attractive) have been extensively studied and are relatively well-understood for interacting membrane surfaces (Barber, 1982; Jia et al., 2014; Puthiyaveetil et al., 2017). As concerns the narrow, densely packed interthylakoidal space in the granum, domination of steric factors, direct protein-protein contacts (Daum et al., 2010; Hind et al., 2014; Albanese et al., 2020) and the role of highly ordered proteinaceous domains (Garab, 2014; Lambrev and Akhtar, 2019) at the interface of adjacent membranes should also be considered, with special regard to the hydration forces.

Presently relatively little is known about the possible role of hydration forces in stabilizing the chloroplast structure. Based on earlier experiments on model phospholipid membranes (Cowley et al., 1978), it is generally assumed that hydration forces, emerging from layers of ordered water molecules, mediate a repulsive interaction between adjacent membrane surfaces. In fact, hydration forces tend to dominate over the van der Waals forces below separations of a few nanometers, as shown by AFM measurements (Israelachvili and Pashley, 1983; Butt, 1991) (see also Puthiyaveetil et al., 2017). Hence, an equilibrium distance in this range is expected in such cases, as, e.g., that of neighboring thylakoid membranes, as well (Chow et al., 2005). Additionally, it can also be argued that oppositely ordered water layers may give rise to an increase of flexibility of the system by facilitating the lateral sliding of neighboring thylakoid membranes (Jia et al., 2014). A massive body of experimental evidences proves that multilevel regulatory mechanisms in the photosynthetic apparatus require a high level of structural and functional plasticity, ensured by a delicate balance between structural stability and flexibility of multilamellar membrane systems (see *Introduction*).

Our experimental results show that chaotropic Hofmeister salts decrease the stability of chloroplast structure at all levels of its hierarchical organization; at the same time the Hofmeister neutral NaCl and the kosmotropic agent (NH_4_)_2_SO_4_ exerted only marginal effects (**Figure 1**). The magnitude and kinetics of the effects are in line with the position of the salts in the Hofmeister series (**Figures 2** and **3**), and can be clearly assigned to the chaotropic effect of SCN^-^ anions, independent or despite the strong effect of high concentrations of Na^+^ cations (**Figure 4**). These marked effects of the Hofmeister salts on the protein macrodomains, and the disassembly of grana (**Figures 6** and **7**) strongly suggest the essential role of hydrophobic/hydrophilic interactions in maintaining the native structure of chloroplasts.

With regard to the sequence of events, it can be seen that the disassembly of granum follows the reverse order when compared to its assembly. Upon treatment by the chaotropic salt NaSCN, the periodicity of the stacked multilamellar array of granum thylakoid membranes was perturbed first, and was lost gradually—as reflected by the gradual weakening and loss of the Bragg diffraction peak (**Figure 7**), as well as by TEM images (**Figure 6**). At high concentrations of NaSCN this occurs virtually as a prompt effect, in less than 2 min (**Figure 7**). It is also interesting to observe that the disassembly of the grana is superimposed on the shrinkage of thylakoid membranes, evidently reflecting an increased stacking due to the high ionic strength/screening effects of Na^+^ ions, possibly also combined with its osmotic effect reducing the lumenal volume. The loss of the periodic order of the thylakoid membranes is followed by the gradual diminishment of the CDψ bands, characteristic of the long-range chiral order of the chromophores in the granum stacks. The presence of CDψ bands, while also depends on the presence of the 3D ultrastructure (Keller and Bustamante, 1986; Garab and van Amerongen, 2009), is diagnostic of the long-range order of the PSII-LHCII supercomplexes. Chaotropic agents gradually dismantle the well-ordered protein macro-arrays; only marginally affecting the LHCII complexes, which, as also shown here (**Figure 8**), play a stabilizing role in the grana. In line with these observations, it was also interesting to observe that tightly stacked microcrystalline lamellar aggregates LHCII (Simidjiev et al., 1997) were not susceptible to Hofmeister effect (**Figure 9**) probably due to lack of structured water between the layers. These aggregates of LHCII with long-range chiral order probably assume the same structure as published by Standfuss et al. (2005) with very close contact between stromal-side residues. Also, in contrast to the loosely stacked LHCII membrane crystals (Hind et al., 2014), which are capable of undergoing dark-reversible light-induced reorganizations, the tightly stacked LHCII lamellar aggregates showed very high stability (Simidjiev et al., 1997).

As for the molecular interpretation of the effects, it can be argued that the highly ordered, structured water-layers between the adjacent granum membrane surfaces are prone to be disturbed by chaotropic anions. As a consequence, the spacer function of the hydration layer is destroyed, yet accompanying with an increased level of fluctuations of the interfacial structure (Dér and Ramsden, 1998; Neagu et al., 2001; Násztor et al., 2016). In fact, SANS signals show that the effects start with a decrease of the periodic order of the stacked membranes. This occurs before the further disassembly of the highly-organized membrane structure take place, as reflected by CDψ, which originate from the long-range chiral order of LHCII and LHCII : PSII supercomplexes in the grana. The most probable scenario for the subsequent events is that, following the collapse of the native interthylakoidal organization, the geometrical constraints, stabilizing the complementary charge patterns, are surpassed by conformational fluctuations. Hence, long-range repulsive forces, due to the overall negative charge of the membranes and the protein residues, start to dominate—loosening the original membrane structure, eventually leading to the destabilization of PSII-LHCII arrays and, albeit only marginally, the supercomplexes themselves.

The results highlight the primary importance of hydration forces in the stabilization of granum stacking inside chloroplasts, and are expected to have general implications for better understanding the structural dynamics of other multifolded membrane organelles, such as mitochondria, as well.

## Data Availability Statement

The original contributions presented in the study are included in the article/supplementary material; further inquiries can be directed to the corresponding authors.

## Author Contributions

LK, AD, and OZ conceived the study. The plants were grown and treatments on isolated thylakoid membranes were applied by OZ, who also carried out the CD spectroscopic measurements. RP carried out the electron microscopy experiments, with the data analyzed by RP, OZ, and GG. SANS measurements were configured by JK (SANS-I) and NS (KWS-2), performed and analyzed by GN, LA, and RÜ with the participation of GG. The paper was written by GG, AD, LK, GN, and OZ, with all authors contributing to the writing.

## Funding

This work was supported by grants of the National Research Development and Innovation Office of Hungary (OTKA KH 124985 and K 128679), and of the Czech Science Foundation (GACR 19-13637S) to GG. GN was, in part, supported by the János Bolyai Research Scholarship of the Hungarian Academy of Sciences and by the ÚNKP-19-4 New National Excellence Program of the Ministry for Innovation and Technology. Infrastructural background and theoretical work was partially supported by the Ministry for National Economy of Hungary through the GINOP-2.3.2-15-2016-00001 program (RP and AD).

## Conflict of Interest

The authors declare that the research was conducted in the absence of any commercial or financial relationships that could be construed as a potential conflict of interest.

The handling Editor declared a past co-authorship with one of the authors LK.
